# Lack of association between platelet indices and disease stage in osteosarcoma at diagnosis

**DOI:** 10.1371/journal.pone.0174668

**Published:** 2017-04-06

**Authors:** Hongtao Li, Yonggang Wang, Zimei Liu, Yuan Yuan, Wentao Huang, Na Zhang, Aina He, Zan Shen, Yuanjue Sun, Yang Yao

**Affiliations:** 1 Department of Oncology, Affiliated Sixth People's Hospital, Shanghai Jiaotong University, Shanghai, People’s Republic of China; 2 Department of Pathology, Affiliated Sixth People's Hospital, Shanghai Jiaotong University, Shanghai, People’s Republic of China; 3 Department of Oncology, Affiliated Sixth People's Hospital South Campus, Shanghai Jiaotong University, Shanghai, People’s Republic of China; Universite de Nantes, FRANCE

## Abstract

**Purpose:**

The purpose of this study was to investigate the relationship between platelet indices [mean platelet volume (MPV), platelet count (PLT), platelet distribution width (PDW) and plateletcrit (PCT)] at diagnosis in osteosarcoma.

**Methods:**

The information of 233 patients with osteosarcoma at diagnosis between 2007 and 2015 was retrospectively reviewed. Clinical parameters such as gender, age, size and site of tumor, and tumor necrosis rate after neoadjuvant chemotherapy were analyzed.

**Results:**

No significant difference was noted in the mean values of MPV, PLT, PDW and PCT among stage I, II and III patients. In localized patients, the median disease-free survival (DFS) values were 42 and 22 months in the PLT<300×10^9^/L and ≥300×10^9^/L groups, respectively, but the difference was not statistically significant (*P* = 0.2611). No difference in the DFS among the three different levels of MPV was observed.

**Conclusion:**

No significantly different platelet indices were noted among the different stages. Although a shorter median DFS was found in localized patients with PLT≥300×10^9^/L, there was still a lack of strong evidence to demonstrate the association between platelet indices and osteosarcoma.

## Introduction

Osteosarcoma (OS) is the most common bone cancer in children and young adults [1, 2]. The 5-year survival in patients without metastasis has improved from 20% to approximately 70% in past decades with a combination of multi-agent neoadjuvant chemotherapy [3, 4]. However, 20% of OS patients with metastatic disease at diagnosis have a low five-year overall survival of approximately 28–33% [5–7].

The correlation between platelet stimulation and cancer spread has been shown in recent years [8–15]. Platelets might promote cancer deterioration by sustaining proliferative signals, resisting cell death, inducing angiogenesis, activating invasion and metastasis, evading immune detection, and supporting cancer stem cells [16]. In OS research, it has been shown that platelets promote the proliferation of OS cell lines through the platelet-derived growth factor-receptor (PDGF-R) signaling axis, and high expression levels of PDGF and PDGF-R in tumor tissue are correlated with tumor progression and shorter disease-free survival in OS patients [17, 18]. These lines of evidence have indicated that there could be a strong link between platelets and OS disease progression.

The mean platelet volume (MPV) is routinely measured by automated common blood count analyzers as a parameter of platelet size [19]. In health individuals, the inverse relationship between MPV and platelet count has been observed by several researches[19]. In cancer patients, one of the most abnormalities is a high platelet count and activated platelets play a pivotal role in cancer metastasis through the release of cytokines and chemokines and the expression of several adhesion receptors [16, 20, 21].

In this study, we retrospectively analyzed 233 OS patients to evaluate whether the platelet indices could be associated with disease stage or the disease-free survival (DFS) of OS patients.

## Methods

### Patients and methods

The clinical records of a series of 233 newly diagnosed osteosarcoma patients referred to the Shanghai Jiao Tong University Affiliated Sixth People’s Hospital (Shanghai, China) between 2007 and 2015 were retrospectively reviewed. The following clinical parameters were recorded in a uniform format on a computer database: gender, age at diagnosis, size and site of tumor, disease stage according to the Enneking surgical staging system, and tumor necrosis rate of surgery samples after neoadjuvant chemotherapy. The laboratory characteristics of blood reports including MPV, PLT, PDW and PCT were performed within 7 days prior to biopsy. Because this study was a retrospective review of the clinical information of anonymous patients and without any treatment intervention, the Ethics Committee of the affiliated Sixth People’s Hospital in Shanghai determined that it was exempt from approval.

### Statistical analysis

All of the parameters were expressed as the means±standard deviation. An independent *t* test was used to compare the parameters of related subjects. All of the clinical factors included were investigated by univariate and multivariate techniques. A comparison of the categorical variables was conducted using the Chi-squared test. Survival curves were estimated using the Kaplan–Meier method and were compared using the log-rank test. A *P*-value less than 0.05 was considered to be statistically significant.

## Results

### Patient characteristics

Of all the analyzed patients, the male-to-female ratio was 66% to 34%, and the mean age was 21 years (range: 7–72 years). There were 10 (4%), 178 (77%) and 45 (19%) patients with stage I, II and III disease, respectively, according to the Enneking surgical staging criteria. The percentage of tumor origin from the femur, tibia and fibula were 61%, 33% and 6%, respectively. Their characteristics are shown in Table 1.

**Table 1:** Basic clinical characteristics of the OS patients.

	Number (%)
Patients	233 (100)
Gender	
female	80 (34)
male	153 (66)
Age (years)	
≤18	130 (56)
>18	103 (44)
Site	
femur	143 (61)
tibia	76 (33)
fibula	14 (6)
Enneking Stage	
I	10 (4)
II	178 (77)
III	45 (19)

### Platelet indices of all included patients

No significant difference distribution of the platelet indices was observed between the metastasis and localized patients by cross table analysis (Table 2). In localized patents, PLT, MPV PDW and PCT also showed no difference between stage I and II (Table 2). There was no significant difference in the mean comparisons of the four indices between the two groups, except a slight higher PLT in age ≤18 years than in age >18 years (244.79±69.85×10^9^/L versus 226.7±67.9×10^9^/L, respectively; *P* = 0.048, Table 3).

**Table 2:** Crosstab analysis of the platelet indices of all 233 osteosarcoma patients.

		All patients (n = 233)			Localized patients (n = 188)		
		Stage I, II	III	*P*	I	II	*P*
PLT (×10^9^/L)	Low	1	0		0	1	
(100–300)	Normal	155	37		8	146	
	High	32	8	0.88	2	31	0.953
MPV (fL)	Low	44	1		1	43	
(9.4–12.6)	Normal	131	30		9	122	
	High	13	14	0.24	0	13	0.338
PDW (fL)	Low	15	2		1	13	
(9.8–16.2)	Normal	140	35		7	134	
	High	33	8	0.71	2	31	0.921
PCT (%)	Low	17	6		0	17	
(0.16–0.38)	Normal	164	36		10	154	
	High	7	3	0.44	0	7	0.462

**Table 3:** Comparison of the platelet indices of all 233 osteosarcoma patients.

		N	MPV (fL)	PLT (×10^9^/L)	PDW (fL)	PCT (%)
Sex	Male	153	10.40±1.93	235.92±64.35	13.44±2.61	0.24±0.07
	Female	80	10.24±1.70	238.68±78.65	12.93±2.59	0.24±0.08
Age(years)	≤18	130	10.35±2.01	244.79±69.85	13.03±2.65	0.25±0.08
	>18	103	10.34±1.64	226.7±67.9 *	13.56±2.54	0.23±0.08
Site	Femur	143	10.42±1.74	234.94±72.24	13.09±2.56	0.24±0.08
	Tibia	76	10.14±2.11	244.82±63.67	13.46±2.68	0.25±0.07
	Fibula	14	10.70±1.36	213.43±68.25	13.90±2.83	0.23±0.06
Metastasis	Yes	45	10.09±1.79	229.63±68.07	13.69±2.33	0.23±0.08
	No	188	10.41±1.87	238.60±69.83	13.16±2.67	0.25±0.07

* *P* = 0.048.

### Platelet indices of localized OS patients

MPV, PLT, PDW and PCT were analyzed in 188 localized patients according to sex, age, tumor size and T stage. A higher PLT in age ≤18 years than in age >18 years was found to be slightly significant in localized patients (247.42±70.95×10^9^/L versus 225.13±65.73×10^9^/L, respectively; *P* = 0.03, Table 4). This result was consistent with the comparison of all 233 patients above.

**Table 4:** Comparison of the platelet indices of localized OS patients according to T stage and tumor size.

		N	MPV (fL)	PLT (×10^9^/L)	PDW (fL)	PCT (%)
Sex	Male	126	13.23±2.73	233.75±67.76	10.47±2.00	0.24±0.07
	Female	62	13.07±2.51	247.98±73.22	10.33±1.56	0.25±0.08
Age (years)	≤18	110	12.91±2.66	247.42±70.95	10.37±2.01	0.25±0.07
	>18	78	13.48±2.65	225.13±65.73^$^	10.52±1.62	0.24±0.08
T	1	16	10.60±1.38	249.31±47.97	13.61±1.84	0.27±0.06
	2	172	10.40±1.90	237.44±71.44	13.14±2.72	0.24±0.08
Size (cm)	<7	66	10.36±1.92	227.24±57.79	13.16±2.68	0.23±0.06
	≥7	122	10.46±1.84	244.51±74.94	13.19±2.66	0.25±0.08

$ *P* = 0.03.

### Disease-free survival (DFS) analysis

**Fig 1:**
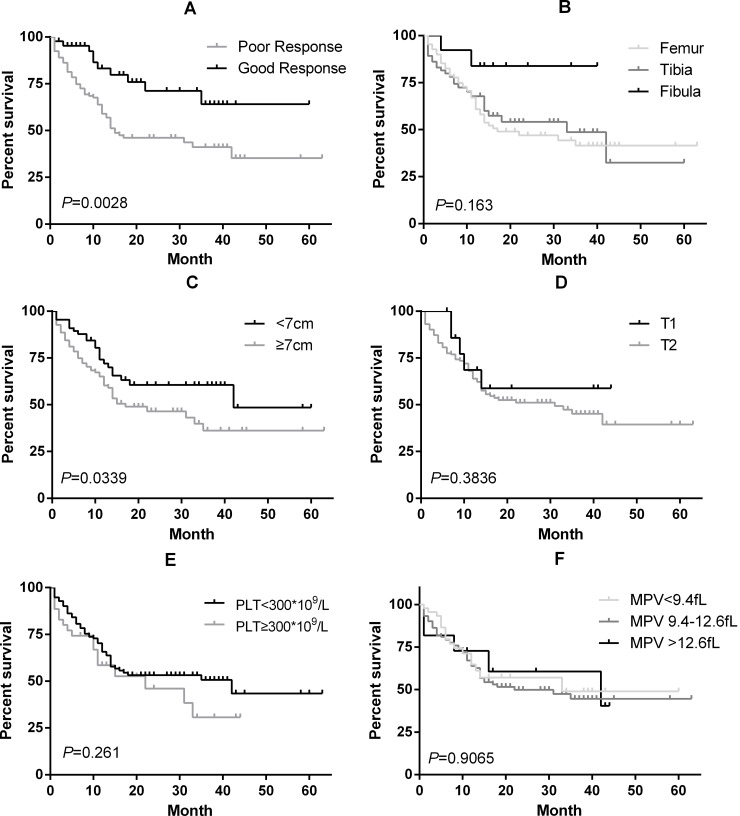
Kaplan-Meier curves for DFS according to the response to neo-adjuvant chemotherapy (A), tumor site (B), tumor size (C), T stage (D), PLT (E), and MPV (F).

Regarding the 188 localized patients, 122 (65%) had a tumor size ≥7 cm, 172 (91%) had a tumor stage at T2, and 45 (22.8%) had a good response after neoadjuvant chemotherapy. The median DFS was statistically significantly longer with a good response (over 60 months with a good response versus 15 months with a poor response; *P* = 0.0028, Fig 1A) and tumor size (42 months with <7 cm versus 17 months with ≥7 cm; *P* = 0.0339, Fig 1C), but not with the tumor site (Fig 1B) or T stage (Fig 1D); an obvious long survival trend was shown for the tumor site at the fibula. Although the median DFS values were 42 and 22 months in the PLT<300×10^9^/L and ≥300×10^9^/L groups, respectively (Fig 1E), the difference was not statistically significant (*P* = 0.2611). No significant difference in the DFS was observed among the different levels of MPV (*P* = 0.9065, Fig 1F).

By including the pretreatment characteristics, the results of univariate and multivariate analyses are shown in Table 5. The factor of tumor size ≥7 cm showed a statistically significantly increased risk of lung metastasis in univariate analyses (Hazard Ratio, HR: 1.68, 95% CI: 1.03±2.76, *P* = 0.039, Table 5) but not in multivariate analyses (HR: 1.64, 95% CI: 0.99±2.71, P = 0.053, Table 5). The levels of PLT, MPV, tumor site, T stage, age and sex showed no statistically significant influence on DFS; a shorter median DFS (22 vs 35 months) was found in localized patients with PLT≥300×10^9^/L than in those with PLT<300×10^9^/L.

**Table 5:** Clinical factors and their effects on disease-free survival by univariate and multivariate Cox proportional hazards regression analysis.

Characteristics		No.	Univariate analysis		Multivariate analysis	
			HR (95% CI)	*P*	HR (95% CI)	*P*
Sex	Male	126	1		1	
	Female	62	1.01 (0.63±1.62)	0.975	0.95 (0.59±1.54)	0.837
Age (years)	≤18	110	1		1	
	>18	78	0.83 (0.52±1.32)	0.429	0.83 (0.51±1.33)	0. 427
Site	Femur	110	1		1	
	Tibia	65	0.94 (0.59±1.52)	0. 809	1.00 (0.62±1.62)	0. 994
	Fibula	13	0.28 (0.068±1.16)	0.079	0.34 (0.08±1.39)	0. 132
Size (cm)	<7	66	1		1	
	≥7	122	1.68 (1.03±2.76)	0.039	1.64 (0.99±2.71)	0.053
T	1	16	1		1	
	2	172	1.48 (0.60±3.67)	0.394	1.53 (0.61±3.83)	0. 332
PLT (×10^9^/L)	<300	153	1		1	
	≥300	35	1.26 (0.73±2.19)	0.410	1.13 (0.64±2.00)	0. 666
MPV (fL)	<9.4	44	1		1	
	9.4–12.6	131	0.84 (0.50±1.41)	0.514	0.88 (0.52±1.48)	0.617
	>12.6	13	0.81 (0.30±2.15)	0.668	0.83 (0.31±2.25)	0.713

## Discussion

This article was the first to delineate the linkage of the platelet indices to disease stage and prognosis of OS. The investigation showed no significant difference in MPV, PLT, PDW and PCT among the different stages. A shorter but statistically insignificant median DFS was found with a high level of PLT.

A higher platelet count or thrombocytosis contributes to cancer metastasis, in agreement with other relevant studies [21–23]. For example, in gastric cancer, the platelet count is higher in metastatic patients than in normal ones (335.84±154.08 vs 247.78±44.74×10^9^/L, respectively) [14] and tends to increase in advanced and node-involved tumors [24]. However, this trend was not observed in the present investigation. The disagreement of the results might be explained by the different origins of sarcoma and carcinoma: sarcoma arises from mesodermal tissue, whereas carcinoma arises from epithelial tissues. Meanwhile, it should be noted that most reports use cut-off values of 350, 400 or 450×10^9^/L, which are much higher than those in this study, and show that an elevated platelet count is correlated with local invasion and metastasis [22, 23, 25]. In this study, there were only 2 localized OS patients with a platelet count higher than 400×10^9^/L, and none of the other 231 patients had a platelet count higher than 350×10^9^/L (data were not shown). The result of the mean PLT stratified for age was consistent with recent reports of a decrease in PLT with increasing age [26].

Tumor-derived interleukin-6 was demonstrated as an underlying mechanism of thrombocytosis in cancer [22], and serum interleukin-6 levels were shown to be 2.4-fold higher in OS than in controls [27, 28]. Moreover, a raised C-reactive protein, the downstream factor of interleukin-6, was associated with poor local control in OS patients[29]. Therefore, it should be inferred that thrombocytosis might be observed in OS similar to other types of cancers. However, no evidence has shown a direct correlation between interleukin-6 and the platelet count in osteosarcoma, indicating that additional deep investigations are needed to elucidate this issue.

Discordant results of the relationship between MPV and different cancers were observed. On the one hand, the MPV was larger in advanced gastric cancers, colon cancer and leukemia than in normal controls, and a positive correlation between larger MPV and tumor-nodule-metastasis (TNM) stage was found [14, 15, 30–32]. On the other hand, a smaller MPV was found in the advanced non-small cell lung cancer (NSCLC) group than in the control group [11], a smaller MPV (<8.50 fL) was shown to predict an unfavorable prognosis in patients with NSCLC [11, 13], and high MPV values were associated with an improved patient survival with cancers [33]. These diversified results revealed that platelet indices might vary according to the different types of cancer. In this analysis, no marked difference in the mean MPV was found, and no good cut-off value was found (data are not shown).

These results might be explained by the evidence that platelet activators in OS patients are locally detected and that there is a different distribution between primary and metastatic tumors. For example, 1) thrombin is one of the most important platelet activators, and the local concentration of thrombin in the bronchoalveolar lavage fluid of 15 OS patients with lung metastasis increased up to more than 100-fold compared with patients without lung metastasis [34]; 2) von Willebrand factor (vWF) is also involved in platelet aggregation and processes critical to hematogenous tumor cell metastasis to the lung, and it was shown that vWF is expressed at higher levels in metastases than in primary tumors [35]. These lines of evidence might indicate that if the tumor load consists mostly of primary tumors, platelets may not be invoked due to insufficient activators derived from a minority of metastatic tumor cells; by contrast, if the tumor load consists mostly of metastatic tumor cells, the platelets are very likely activated.

Although this is the first article focusing on platelets in OS, drawbacks still exist: (1) the overall survival was not analyzed because of the lack of sufficient data; (2) Interleukin-6, lactate dehydrogenase, alkaline phosphatase and subtypes of OS were not considered in this analysis; and (3) changes in the platelet indices before and after neoadjuvant chemotherapy were not measured to better understand the relationship between these two parameters.

In conclusion, there was no remarkable difference in the platelet indices among the different stages of OS. Although a shorter but statistically insignificant median DFS was found in localized patients with PLT≥300×10^9^/L, there was still a lack of strong evidence to demonstrate the association between platelet indices and osteosarcoma. The potential role of platelets in OS remains to be investigated at a broader and deeper level to verify the possible clinical significance of this finding.

## Supporting information

S1 Dataset Original dataset of anonymous OS patients. (XLSX)
